# Natural language processing for triage of cerebral large-vessel occlusion

**DOI:** 10.1055/s-0045-1813238

**Published:** 2025-12-02

**Authors:** João Brainer Clares de Andrade, José Marcio Duarte, Thales Pardini Fagundes, Thiago Bulhões da Silva Costa, Paulo B. Paiva, André Shimaoka, Antonio C. da Silva Junior, Evelyn de Paula Pacheco, Sophia Oliveira Querobin, Marialdo Augusto Cordeiro de Souza Junior, Eduardo Saucedo Lage, Gisele Sampaio Silva

**Affiliations:** 1 Universidade Federal de São Paulo, Departamento de Informática em Saúde, São Paulo SP, Brazil.; 2 Universidade Federal de São Paulo, Departamento de Neurologia, São Paulo SP, Brazil.; 3 Hospital Israelita Albert Einstein, Centro de Ensino e Pesquisa Albert Einstein, São Paulo SP, Brazil.; 4 Instituto Tecnológico de Aeronáutica, São José dos Campos SP, Brazil.; 5 Hospital de Amor de Barretos, Serviço de Neurologia, Barretos SP, Brazil.; 6 AMIL Sistema de Saúde, Serviço de Teleneurologia, São Paulo SP, Brazil.; 7 Centro Universitário São Camilo, Faculdade de Medicina, São Paulo SP, Brazil.

**Keywords:** Artificial Intelligence, Mass Screening, Natural Language Processing, Stroke

## Abstract

**Background:**

Timely identification of large-vessel occlusion (LVO) in ischemic stroke is essential for optimizing prehospital triage and enabling rapid mobilization of thrombectomy-capable teams. Traditional LVO screening tools are often lengthy and reliant on neurological examination skills that may be inaccessible to nonspecialists.

**Objective:**

To assess the ability of large language models (LLMs) to detect LVO using only free-text summaries, with or without National Institutes of Health Stroke Scale (NIHSS) data, in a national teleneurology service.

**Methods:**

We conducted a retrospective analysis of 2,887 suspected stroke cases across 21 spoke hospitals within a national TeleStroke network. Neurologist-authored case summaries were processed using natural language processing techniques, including text embedding and supervised machine learning classification. Contextual LLMs (BERTimbau, BioBERTpt, GPorTuguese-2) were evaluated with five algorithms. The Bootstrap method was employed to mitigate class imbalance, with performance averaging over 100 iterations.

**Results:**

Of 1,060 cases included in the final dataset, 143 had confirmed proximal occlusions. Median Alberta Stroke Program Early CT Score (ASPECTS) was 9 and mean National Institutes of Health Stroke Scale (NIHSS) was 5.4 ± 2. AdaBoost paired with BioBERT yielded the highest accuracy (89.82%), precision (98.37%), and AUC (89.86%). Incorporating NIHSS as a numerical feature improved recall (87.60% with multilayer perceptron) and F1-score (89.05% with Dense Neural Network). BioBERT consistently outperformed other models, regardless of NIHSS inclusion.

**Conclusion:**

The LLM-based models demonstrated strong performance in identifying LVO using routine clinical narratives. These findings support the integration of NLP and ML in TeleStroke systems and underscore the need for further validation across larger, multilingual datasets to ensure generalizability and clinical applicability.

## INTRODUCTION

Large-vessel occlusion (LVO) strokes account for 10 to 30% of stroke cases admitted to hospitals. 1 These cases can only be reliably identified after imaging or sonographic evaluation and require prompt treatment, close monitoring, and complex clinical management. 1 These are associated with poorer functional outcomes, higher hospitalization costs, and increased likelihood of permanent disability. 2 They may be treated up to 24 hours after onset, provided that specific trial criteria are met, offering substantial clinical benefit and cost-effectiveness—even in developing countries. 3 As such, LVO stroke cases demand rapid identification and triage during the initial moments of care, particularly considering access to mechanical thrombectomy is often limited in developing nations and remote areas, necessitating patient transfer to specialized centers. 4

With the expansion of stroke care pathways and the adoption of new protocols extending treatment windows, TeleStroke networks have emerged as crucial resources in managing the stroke care continuum. 5 These networks guide treatment decisions and coordinate patient transfer and regulation within healthcare systems. Optimizing and improving the timing of diagnosis and transfer remains a key objective for TeleStroke networks, ensuring the best possible clinical outcomes. 6

However, the triage process continues to pose challenges. 7 8 Clinicians at stroke hospitals or in prehospital mobile units may lack familiarity with LVO screening scales, which often have limited accuracy, can be complex, and may require significant time to apply. 9 Implementing more effective strategies for its identification could significantly reduce triage times, enabling faster treatment initiation or transfer of patients to specialized centers equipped to provide advanced care. 10

Natural language processing (NLP) is arising as a valuable tool in the stroke care continuum. 11 Its applications include mining data from electronic health records to assess clinical scales, which includes identifying findings in neuroimaging and generating large datasets for quality-of-care metrics. 12 Despite its potential, NLP initiatives on stroke triage remain scarce. However, NLP could serve as a simultaneous resource within TeleStroke workflows, as it does not require additional scale completion or form-filling. 12 The repetition of patterns in the signs and symptoms of patients with LVO, as reported by numerous authors, presents a promising opportunity for NLP to screen these cases. 13

We aimed to describe the development and validation of a NLP algorithm capable of identifying patients with or without LVO, as confirmed by computed tomographic angiography (CTA), from a national hospital-based TeleStroke network, solely through evaluating clinical and demographic data.

## METHODS

### Study design

The study was designed as a retrospective analysis, using data from consecutive cases managed by a national TeleStroke service, covering 21 spoke hospitals in Brazil, from March 2022 to 2024.

### Data source and participant centers

The input for the NLP model consisted of case summaries written by neurologists from the TeleStroke service. These summaries were based on information provided by the spoke hospital physician. The physical exams were conducted collaboratively via video or independently by the nonneurologist physician at the spoke hospital. To ensure data privacy, all patient and assisting team identifiers were removed before processing the text through the NLP algorithm.

As per the institutional stroke protocol, all patients with suspected ischemic stroke (IS) and National Institutes of Health Stroke Scale (NIHSS) > 0 points and Alberta Stroke Program Early CT Score (ASPECTS) between 1 and 10 were eligible for CTA imaging. A centralized neuroradiology team reported the imaging results within 20 minutes.

Participant spoke hospitals were equipped with 24/7 generalist physicians, standardized acute stroke protocol, 24/7 availability of CT and CTA imaging, and centralized radiology reporting. Suspected stroke cases were assessed by teams with formal training and expertise in neuroradiology. For the CTA, the site of intracranial occlusion was defined as the most proximal site of occlusion, including the internal carotid artery (ICA) or first segment of the middle cerebral artery (MCA-M1). The presence of M2 occlusion was an exclusion criterion. Given the absence of treatment recommendations 14 for M2 occlusions in the guidelines available at the time of our algorithm's development, we deemed the identification of such cases clinically irrelevant and, therefore, excluded them.

The NIHSS's subitems, type of treatment, and clinical outcomes were not collected, due to the nature of the data source.

### Inclusion and exclusion criteria

Consecutive suspected stroke cases with documented neurological examination, NIHSS scores, and ASPECTS within the defined ranges were eligible for analysis. On the other hand, cases missing descriptions of the neurological exam, NIHSS, or ASPECTS, as well as eligible cases for CTA without the imaging, were excluded in the final analysis. Patients with M2 occlusions were also excluded.

### Natural language processing algorithm

The algorithm was created based on data preprocessing, embedding generation, application of the Bootstrap method, and implementation of machine learning (ML) algorithms for supervised classification processes. These stages created a systematic framework that optimized the classification process and enhanced the overall effectiveness of the model.

**Supplementary Material 1–Figure S1** (available at https://www.arquivosdeneuropsiquiatria.org/wp-content/uploads/2025/09/ANP-2025.0149-Supplementary-Material-Figure-S1.pptx ) depicts the preprocessing phase and the generation of embeddings. Initially, the text undergoes a cleaning process wherein all words are converted to lowercase, and punctuation marks and special characters (e.g., “#!? < > ”) are removed. This step ensures that only textual characters are provided to the Large Language Model (LLM). Subsequently, a Python (Python Software Foundation) function was implemented to eliminate stopwords using the Natural Language Toolkit (NLTK). The purpose of this step is to remove words that do not contribute to the semantic meaning of the text.

**Figure 1:**
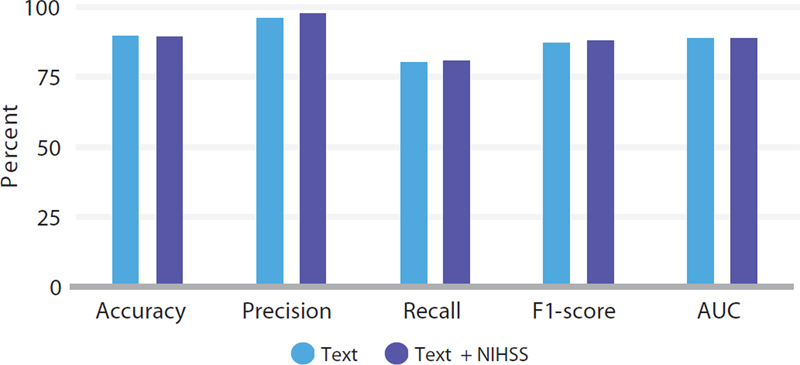
Results of BioBERT plus AdaBoost using text only or text + National Institutes of Health Stroke Scale.

The filtering process includes removing abbreviations, specific Brazilian Portuguese nouns, adverbs, prepositions, articles, and other nonessential words. This preprocessing ensures the text is refined and optimized for embedding generation.

The subsequent processing phase is tokenization, which prepares the input for a language model. Tokenization involves segmenting text strings into subword token strings and converting these tokens into integer representations.

Three contextual LLMs designed for Brazilian Portuguese were employed to transform words into embeddings. The first was the BERT-base-portuguese-cased (BERTimbau Base), 15 a BERT model for Brazilian Portuguese trained on millions of web pages from the Brazilian Web as Corpus (BRWAC). The second model, BioBERTpt (all), is a BERT-based model trained on Portuguese clinical notes 16 and biomedical corpora, including PubMed and Scielo. Lastly, Portuguese GPT-2 small (GPorTuguese-2) was trained on Portuguese Wikipedia.

While standard NLP preprocessing steps often include removal of stop words and punctuation. Lee et al. 17 have shown that preserving these elements can improve model performance in biomedical and clinical NLP tasks. Future iterations of our model will assess the impact of maintaining such linguistic structures on classification accuracy.

### Bootstrap

The Bootstrap method is illustrated in **Supplementary Material 2–Figure S2** (available at https://www.arquivosdeneuropsiquiatria.org/wp-content/uploads/2025/09/ANP-2025.0149-Supplementary-Material-Figure-S2.pptx ) After generating the embeddings, undersampling was managed using the APRICOT tool (Bonterra), which employs submodular optimization to summarize large datasets into representative subsets. This step was necessary because our data set is unbalanced in the final sample, comprising 143 positive and 917 negative cases of LVO. To address this imbalance, 70% of the samples from LVO-negative and -positive were randomly selected using the Resample function (143 × 0.7).

**Figure 2:**
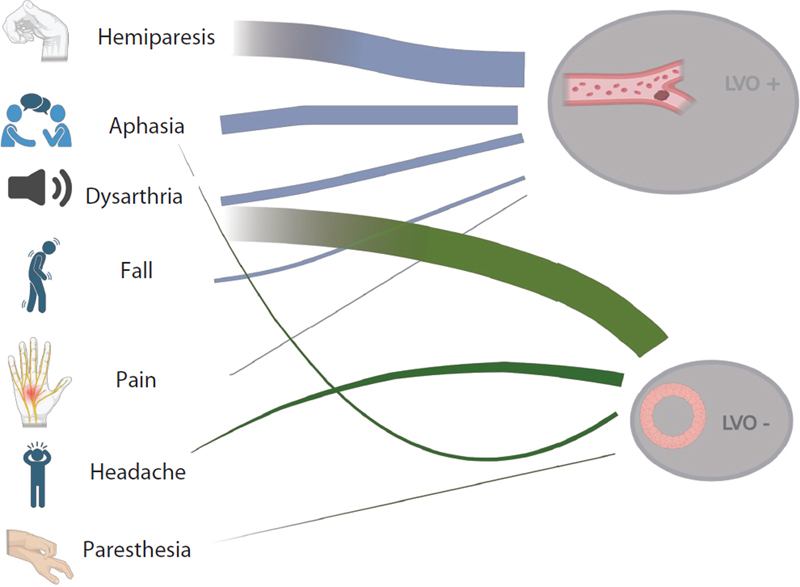
Most cited words between large-vessel occlusion positive and negative cases.

The sampling was conducted with replacement, and the remaining samples not included in the training set were allocated to the test set. Subsequently, the training and test samples for LVO-negative and -positive cases were concatenated. The model was then trained and evaluated for predictions. This process was repeated 100 times, and the mean and standard deviations (SD) of the results were computed.

There were five algorithms employed to train supervised classification models: 19 multilayer perceptron (MLP), k-nearest Neighbors (kNN), Random Forest (RF), AdaBoost, and a Dense Neural Network (NN). The MLP, kNN, and RF algorithms were implemented using the Python library sci-kit-learn, while the Dense NN was implemented using Keras, a deep learning API. The parameter *k* for kNN was determined through cross-validation and varied based on the attributes and large language models (LLMs), as shown in **Supplementary Material 3, Table S1** (available at https://www.arquivosdeneuropsiquiatria.org/wp-content/uploads/2025/09/ANP-2025.0149-Supplementary-Material-3.docx ).

The RF algorithm was configured with the following parameters: max_depth = 20, n_estimators = 100, and max_features = 1. For AdaBoost, the RF algorithm served as the base estimator, with n_estimators = 400 and a learning rate 0.7. The MLP was set with alpha = 1 and max_iter = 1,000. The Dense NN was configured using the Sequential API with two hidden layers, each employing the ReLU activation function and a dropout rate 0.2. The output layer used a sigmoid activation function, with binary cross-entropy as the loss function and the Adam optimizer.

As a frequency analysis method, we adopted the attention-weighted token analysis, which is widely used in clinical text mining to discern salient terms. This would improve transparency and reproducibility of “feature importance” attribution. 20

### Standard protocol approvals, registrations and data availability

The Institutional Review Board (IRB) for the Hospital Samaritano de São Paulo, CAAE 78600924.1.0000.5487, approved the present study. All the national ethical requirements were observed to support the research. All data used in the analysis are presented in the tables and figures. Anonymized data, study protocol, statistical analysis plan, and informed consent forms will be shared by request from other investigators after ethics approval.

## RESULTS

During the study period, 2,887 consultations were recorded with suspected ischemic stroke. A total of 1,060 cases were included in the final analysis, based on the criteria. Among these, 143 cases were confirmed to have proximal occlusion (carotid siphon and/or M1 segment of the middle cerebral artery). The median ASPECTS was 9 [9, 10], and the NIHSS on admission had a mean score of 5.4 ± 2 points. Age and sex were not considered in the final dataset to avoid introducing bias into the natural language processing algorithm.

The results of LLMs and ML methods applied to free text only are presented in Table 1 . The AdaBoost method, when combined with the BioBERT LLM, demonstrated superior performance with an Accuracy of 89.82%, Precision of 97.94%, F1-score of 88.83%, and AUC of 89.82%, when compared with other methods. When paired with BioBERT, the MLP method achieved the best Recall at 82.00%, and the RF algorithm delivered results comparable to those of AdaBoost. Figure 1 depicts results of BioBERT plus AdaBoost using Text only or Text plus NIHSS.

**Table 1:** Average (% ± SD) from LLM results and ML methods applied on free text only samples

	Acc	Prec	Recall	F1	AUC
BERTimbau (MLP)	75.84 ± 3.06	75.03 ± 4.00	78.20 ± 5.89	76.40 ± 3.33	75.84 ± 3.08
BERTimbau (kNN)	68.15 ± 3.93	66.61 ± 4.53	72.75 ± 6.72	69.33 ± 4.01	68.20 ± 3.92
BERTimbau (RF)	74.51 ± 3.45	73.53 ± 5.08	77.65 ± 5.77	75.28 ± 3.21	74.59 ± 3.42
BERTimbau (AdaBoost)	76.53 ± 3.73	74.73 ± 5.59	81.19 ± 5.95	77.54 ± 3.39	76.64 ± 3.68
BERTimbau (NN)	74.01 ± 3.84	71.31 ± 5.25	81.64 ± 8.86	75.62 ± 4.29	74.07 ± 3.79
BioBERT (MLP)	85.78 ± 2.88	88.96 ± 4.90	**82.00 ± 4.14**	85.20 ± 2.96	85.79 ± 2.86
BioBERT (kNN)	87.08 ± 2.82	92.12 ± 5.39	81.57 ± 4.07	86.35 ± 2.78	87.07 ± 2.80
BioBERT (RF)	89.54 ± 1.81	97.05 ± 3.09	81.58 ± 3.69	88.55 ± 2.06	89.51 ± 1.81
BioBERT (AdaBoost)	**89.82 ± 2.05**	**97.94 ± 2.23**	81.41 ± 4.14	**88.83 ± 2.41**	**89.82 ± 2.01**
BioBERT (NN)	86.93 ± 3.13	92.67 ± 6.36	81.14 ± 4.76	86.25 ± 2.92	87.00 ± 3.11
GPorTuguese-2 (MLP)	83.70 ± 2.14	88.09 ± 3.94	78.12 ± 4.75	82.64 ± 2.49	83.70 ± 2.12
GPorTuguese-2 (kNN)	84.16 ± 2.06	88.79 ± 4.20	78.65 ± 4.60	83.24 ± 2.40	84.19 ± 2.07
GPorTuguese-2 (RF)	84.99 ± 2.40	90.66 ± 3.62	78.08 ± 4.70	83.78 ± 2.90	84.97 ± 2.43
GPorTuguese-2 (AdaBoost)	85.22 ± 2.18	90.75 ± 3.23	78.61 ± 4.50	84.13 ± 2.64	85.23 ± 2.18
GPorTuguese-2 (NN)	83.25 ± 3.05	86.58 ± 5.65	79.41 ± 5.34	82.59 ± 3.16	83.27 ± 3.03

Abbreviations: AUC, area under the curve; RF, random forest; kNN, k-nearest neighbors; LLM, large language model; ML, machine learning; MLP, multilayer perceptron; NN, neural network; SD, standard deviation.

Note: The best-performing values are in bold.

Table 2 outlines the outcomes of LLMs and ML methods applied to free-text data paired with numerically assessed NIHSS scores. Once again, the AdaBoost method with BioBERT outperformed other approaches in terms of Accuracy (89.82%), Precision (98.37%), and AUC (89.86%). The MLP method with BioBERT yielded the highest Recall (87.60%), while the Dense NN achieved the best F1-score (89.05%).

**Table 2:** Average (% ± SD) from LLM and ML methods applied on free-text samples plus NIHSS feature

	Acc	Prec	Recall	F1	AUC
BERTimbau (MLP)	77.38 ± 3.18	76.31 ± 4.91	79.80 ± 5.01	77.83 ± 3.07	77.40 ± 3.17
BERTimbau (kNN)	71.77 ± 3.22	73.15 ± 4.32	69.94 ± 6.63	71.26 ± 3.89	71.76 ± 3.21
BERTimbau (RF)	74.05 ± 3.34	73.48 ± 4.87	76.76 ± 6.79	74.79 ± 3.44	74.10 ± 3.34
BERTimbau (AdaBoost)	76.83 ± 3.85	74.79 ± 5.30	81.73 ± 6.10	77.86 ± 3.65	76.93 ± 3.83
BERTimbau (NN)	77.22 ± 3.30	76.25 ± 4.58	79.78 ± 8.03	77.63 ± 4.00	77.25 ± 3.31
BioBERT (MLP)	88.85 ± 1.98	90.09 ± 3.87	**87.60 ± 4.07**	88.70 ± 2.08	88.85 ± 1.97
BioBERT (kNN)	88.02 ± 2.32	90.84 ± 3.94	84.94 ± 3.6 **7**	87.69 ± 2.33	88.04 ± 2.31
BioBERT (RF)	89.11 ± 1.94	97.17 ± 2.79	80.78 ± 3.77	88.14 ± 2.25	89.17 ± 1.92
BioBERT (AdaBoost)	**89.82 ± 1.84**	**98.37 ± 2.04**	81.13 ± 3.89	88.85 ± 2.19	**89.86 ± 1.80**
BioBERT (NN)	89.42 ± 2.18	93.05 ± 4.02	85.62 ± 4.0 **1**	**89.05 ± 2.21**	89.47 ± 2.10
GPorTuguese-2 (MLP)	84.07 ± 2.23	85.65 ± 4.29	82.44 ± 5.59	83.79 ± 2.48	84.09 ± 2.24
GPorTuguese-2 (kNN)	80.01 ± 3.29	79.48 ± 4.40	81.39 ± 6.04	80.23 ± 3.57	80.06 ± 3.31
GPorTuguese-2 (RF)	84.79 ± 2.30	90.92 ± 3.47	77.51 ± 4.67	83.55 ± 2.78	84.81 ± 2.28
GPorTuguese-2 (AdaBoost)	84.89 ± 2.16	91.30 ± 3.36	77.44 ± 4.85	83.65 ± 2.66	84.91 ± 2.11
GPorTuguese-2 (NN)	84.17 ± 3.00	84.07 ± 4.99	84.83 ± 5.98	84.21 ± 3.21	84.15 ± 3.02

Abbreviations: AUC, area under the curve; RF, random forest; kNN, k-nearest Neighbors; LLM, large language model; ML, machine learning; MLP, multilayer perceptron; NIHSS, National Institutes of Health Stroke Scale; NN, neural network; SD, standard deviation.

Note: The best-performing values are in bold.

In **Supplementary Material 3, Table S2** highlights the differences between the analyses shown in Tables 1 and 2 . The addition of the NIHSS feature led to notable improvements in Accuracy, Precision, and AUC for the combination of BERTimbau and kNN. Regarding Recall and F1-score, BioBERT paired with MLP showed the greatest improvement. For AdaBoost using BioBERT, the addition of the NIHSS feature resulted in minimal differences compared to the model utilizing only text data. The inclusion of the numerical NIHSS feature proved particularly important for the Recall metric, which is crucial for distinguishing true positives from false negatives. The MLP method with BioBERT achieved the highest Recall value (87.60%).

Although BioBERT combined with AdaBoost consistently delivered the best results across most metrics, the addition of the NIHSS feature resulted in only marginal differences in scenarios featuring text alone.

Descriptive accuracies of published clinical scales and our algorithm is available in Table 3 . 8 21 22 23 24 25 26 27 28 29 30 31 32 33 34 Our model achieved an AUC of 0.898, which compares favorably with most of the listed scales. However, a head-to-head evaluation was not conducted using the same dataset for both the NLP model and these scales.

**Table 3:** Comparing accuracy of large vessel occlusion screening scales

Scale or Tool	Variables	AUC ^1^	AUC ^2^
NIHSS 21	Consciousness and responsiveness, gaze, visual fields, facial palsy, motor strength, limb ataxia, sensory, language, dysarthria, and extinction and inattention (neglect).	0.86	0.86 22
FAST-ED 8 22	Facial palsy, arm weakness, speech changes, eye deviation, and denial/neglect	0.81 8	0.68 23
mG-FAST 23	Gaze deviation, facial palsy, arm weakness, and speech	0.89	NA
RACE 24	Following commands (close your eyes, make a fist - only scored if right hemiparesis), gaze deviation, facial palsy, motor (arm and leg), and neglect (only scored if left hemiparesis)	0.85	0.83 22
CP-SSS 25 26	Conjugate gaze deviation, questions (patient age and current month), commands (close eyes and open/close hand), and motor (hold either or both arms up for 10 seconds).	0.67 27	0.85 26
LVO score 21	NIHSS and relative attenuation value of middle cerebral artery on non-contrast CT.	0.91	NA
HAS 27	Hyperdense artery sign on non-contrast CT.	0.5	NA
LAMS 28	Facial palsy, handgrip, arm weakness	0.89 29	0.72 30
Pomona 29	Gaze, expressive aphasia, and neglect	0.79	NA
PASS 30	Consciousness and responsiveness (month/age), gaze, and arm weakness	0.74	NA
SAVE 31	Speech, arm (motor), visual fields, gaze	0.79	NA
C-STAT 32	Gaze (≥1 point on NIHSS item), questions (patient age and current month) and does not follow one of 2 commands) commands (close eyes, opening and close hand), motor (hold either or both arms up for 10 seconds before falls to bed).	Sensitivity 71% (95% CI 29–96)	0.75 22
VAN 33	Motor (arms), vision, aphasia and neglect	0.92	NA
3I-SS 34	Facial palsy, arm weakness, language	0.93	NA
**Our tool**	Natural language	0.898	NA

Abbreviations: 3I-SS, 3-Item Stroke Scale; ACT-FAST, Ambulance Clinical Triage and Field Assessment Stroke Triage; AUC, area under the curve; C-STAT, Cincinnati Stroke Triage Assessment Tool; CI, confidence interval; CP-SSS, Cincinnati Prehospital Stroke Severity Scale; CT, computed tomography; FAST-ED, Field Assessment Stroke Triage for Emergency Destination; HAS, hyperdense artery sign; LAMS, Los Angeles Motor Scale; LVO, large vessel occlusion; NA, bot available; NIHSS, National Institutes of Health Stroke Scale; PASS, Prehospital Acute Stroke Severity; RACE, Rapid Arterial Cclusion Evaluation Scale; SAVE, speech arm vision eyes; VAN, stroke vision, aphasia, neglect.

Notes: ^1^ From the derivation group; ^2^ From the validation group.

The most cited words from each clinical group were represented in Figure 2 . In the updated word-frequency analysis, LVO-positive narratives were dominated by cortical motor and language terms—hemiparesis (highest), aphasia, dysarthria, and hemiplegia—with additional entries for paresis and event descriptors, such as fall and pain. In contrast, LVO-negative narratives most frequently featured dysarthria (highest), followed by hemiparesis/paresis, and nonspecific complaints, such as headache and paresthesia. Aphasia also appeared but at a lower rank than in LVO-positive cases. These lexical patterns are consistent with the enrichment of focal language and severe motor deficits in LVO, supporting their inclusion as discriminative features in our model.

## DISCUSSION

Our results highlight the potential of NLP algorithms, specifically those leveraging large language models and machine learning, in accurately identifying large vessel occlusion cases. 12 The performance metrics achieved here, particularly with the combination of BioBERT and AdaBoost, demonstrated remarkable accuracy and precision, outperforming most clinical screening scales currently used for LVO detection.

Our algorithm enhances a wide range of applications, potentially marking a significant breakthrough in georeferencing, triage, and the referral of patients with acute stroke, where timely and accurate decision-making is critical. 35 36 The findings suggest that the developed algorithm can be integrated into TeleStroke services, for instance, to streamline diagnostics and expedite the referral of patients to specialized centers.

Therefore, whether processing the clinical note from the consultation or transcribing audio conversations between spoke and hub hospitals, our algorithm can enhance LVO case identification and streamline referral workflows effectively. According to the literature, 37 this resource may reduce unnecessary interhospital transfers, the algorithm may generate cost savings estimated at $800 to 1,200 per avoided case, exceeding traditional teleconsultation margins by 20 to 30%, with a projected break-even point reached after approximately 90 to 120 triage assessments for suspected LVO cases.

A notable advantage of the proposed algorithm is its independence from systematic neurological examination. However, a central methodological limitation is that the model was trained on summaries authored by expert stroke neurologists, who—despite not being physically present—often elicit and integrate details beyond what is spontaneously documented by nonspecialists. This likely explains the prominence of terms such as aphasia, hemiparesis, and falls, which reflect cortical and motor deficits typically emphasized in validated stroke scales. While this enhances signal quality, it limits ecological validity in prehospital or early hospital settings, where documentation is sparser and less specialized. Unlike traditional screening scales that rely on detailed physical assessments, our approach analyzes textual data to infer the likelihood of LVO. This feature allows the algorithm to be widely applicable, even in prehospital settings, such as by paramedics, when full neurological evaluations may not be feasible. 12 Importantly, the algorithm demonstrated robust accuracy even without incorporating NIHSS scores as a numerical feature, underscoring its effectiveness in diverse clinical and operational contexts.

The relatively low median NIHSS score observed among patients with confirmed proximal occlusion likely reflects the specific clinical profile of our cohort, which consisted predominantly of mild-to-moderate strokes triaged in a national TeleStroke network. Several factors may contribute to this finding, including the early timing of assessments, potential underestimation by generalists in preconsultation evaluations, and limited NIHSS documentation of deficits not easily accessed via telemedicine—such as neglect or subtle hemiparesis. 38 Moreover, some patients may have presented with isolated cortical signs (e.g., aphasia or gaze deviation) that contribute fewer points to the total NIHSS, despite their diagnostic relevance for LVO. 39 These nuances highlight NLP's potential to capture clinically meaningful patterns beyond traditional score thresholds.

Interestingly, the algorithm identified the most frequently cited words in LVO-positive cases, which overlap with the terms present in several established clinical scales. 35 However, certain terms commonly used in these scales and reported in the literature, 40 such as conjugate gaze deviation, facial paralysis, and extinction/neglect, were not top ranked by the model. This discrepancy may reflect inherent limitations of conducting neurological examinations via telemedicine. Unlike physical assessments performed in person, video-based evaluations may not reliably capture subtle clinical signs. These findings underscore the need to adapt diagnostic tools to the constraints of remote settings, ensuring their reliability and applicability. The literature cites a high value of clinical judgment 41 by well experienced neurologists; however, this kind of professional is not widely available, even in well-structured TeleStroke services. Moreover, our sample predominantly includes patients with low neurological severity. This lower incidence of cortical deficits may explain their reduced representation in our algorithm.

Effective deployment of AI-based tools in telemedicine workflows hinges on balancing computational efficiency and clinical responsiveness. In time-sensitive conditions, such as stroke triage, low-latency inference is critical. Cloud-based architectures provide scalable resources and centralized model updates but depend on stable internet connectivity, which may be unreliable in rural or bandwidth-limited regions served by many spoke hospitals in TeleStroke networks. 42 43 Conversely, edge computing—where models are deployed on local servers or mobile devices—offers real-time processing with minimal latency, but requires optimized, lightweight model architectures and sufficient local hardware capacity. 43

Given the high performance of the BioBERT plus AdaBoost model in our study, implementing such solutions at the edge would necessitate significant model compression or distillation techniques to maintain performance without exceeding memory constraints. Hybrid strategies—where an initial screening occurs at the edge and uncertain or complex cases are escalated to cloud-based processing—may represent a feasible model for broader telehealth adoption. 42 43 These trade-offs must be considered when scaling NLP applications like ours to remote prehospital units or low-resource healthcare settings.

This study is not without limitations. One major constraint was the relatively small number of LVO-positive cases, which may limit the generalizability of the findings; this raises concerns about overfitting, despite our use of bootstrap resampling and multiple iterations. Validation in larger and more diverse datasets is essential to ensure robustness.

Additionally, the textual patterns analyzed were specific to a single telemedicine network, albeit involving 21 units, and the algorithm was tested exclusively in Brazilian Portuguese.

The potential for bias due to inconsistencies in NIHSS scoring and anamnesis conducted by nonneurologist physicians must also be acknowledged. Furthermore, model validation relied on extrapolations and imputations using bootstrap methods, which, while methodologically sound, introduce their own set of uncertainties. Moreover, we did not have sufficient data in our dataset to conduct an assessment of scales previously published in the literature; this validation resource could be useful in corroborating the performance of our algorithm.

Finally, although the models are intended for use in settings without neurologists—such as spoke hospitals or prehospital mobile units—they were trained on case summaries authored by TeleStroke neurologists. While these summaries are based on information provided by nonneurologist personnel, they likely reflect neurologists' interpretive expertise, which may introduce bias toward specialist language and clinical framing not typical of documentation from nonspecialists. This could limit generalizability to real-world prehospital settings. A more suitable approach would involve training the models on texts directly generated by nonneurologist physicians and prehospital providers who operate within these environments.

In conclusion, the present work reinforces the potential of large language models not only for classification tasks but also as generative tools capable of identifying relevant clinical features and informing the development of simplified, data-driven triage scales. By highlighting which terms and patterns most consistently align with LVO, our findings suggest a path toward NLP-assisted clinical reasoning.

Future studies may include the dynamic generation of stroke severity summaries, tailored decision support in low-resource settings, or even real-time feedback systems for nonspecialist clinicians—especially when neurological expertise is unavailable. To support such advances, greater clarity on the origin, structure, and granularity of the source notes will be essential for readers to appraise the generalizability and translational value of these tools.

### Declaration of generative AI and AI-assisted technologies in the writing process

During the preparation of the present work, the authors used ChatGPT 4.0 (OpenAI, Inc.) in order to review grammar. After using this tool/service, the authors reviewed and edited the content as needed and take full responsibility for the content of the publication.
